# Examining the effects of teacher self-compassion, emotion regulation, and emotional labor strategies as predictors of teacher resilience in EFL context

**DOI:** 10.3389/fpsyg.2023.1190837

**Published:** 2023-07-21

**Authors:** Yan Hu

**Affiliations:** School of Maxism, Sichuan International Studies University, Chongqing, China

**Keywords:** self-compassion, emotion regulation, emotional labor strategies, resilience, EFL teachers

## Abstract

**Introduction:**

This study investigates the effects of teacher self-compassion, emotion regulation, and emotional labor strategies on teacher resilience in the English as a foreign language (EFL) context. The study aims to understand the relationships between these variables and their potential implications for promoting teacher resilience.

**Methods:**

A sample of 711 Chinese EFL teachers participated in the study. Confirmatory factor analysis (CFA) was conducted to assess the psychometric properties of the instruments used to measure teacher self-compassion, emotion regulation, emotional labor strategies, and teacher resilience. Structural equation modeling (SEM) was employed to examine the relationships between these variables.

**Results:**

The results of the study revealed that teacher self-compassion and emotional labor strategies had direct positive effects on teacher resilience. Specifically, higher levels of self-compassion and effective use of emotional labor strategies were associated with greater teacher resilience. Furthermore, teacher emotion regulation was found to indirectly predict teacher resilience through the mediation of emotional labor strategies. This suggests that the ability to regulate emotions influences the adoption of effective emotional labor strategies, which in turn contributes to higher levels of teacher resilience.

**Discussion:**

The findings of this study highlight the importance of teacher self-compassion, emotion regulation, and emotional labor strategies in promoting teacher resilience in the EFL context. Interventions aimed at enhancing teacher emotional regulation skills and fostering self-compassion may have significant implications for supporting teachers in managing the demands and challenges of their profession, ultimately enhancing their resilience. These findings contribute to the understanding of factors that can promote teacher resilience and inform the development of targeted interventions in the EFL context.

## Introduction

Teaching is a difficult and labor-intensive profession, due to the demands of responsibility and its foundation in service-providing, and that is why educators are regarded as the primary stakeholders in the challenging field of language instruction (Mercer, 2020). In the context of teaching English as a foreign language (EFL), educators encounter unique obstacles stemming from language and cultural differences. These challenges not only heighten the demands on their emotional and cognitive resources but also pose distinct difficulties compared to other professions (Chen and Goh, 2011; Derakhshan et al., 2022; Liu et al., 2023). Unlike many occupations, second/foreign language teachers have responsibilities that transcend the boundaries of the classroom (Johnston, 1997). Their duties encompass assigning meaningful homework, providing valuable feedback, and monitoring students’ progress (Kalaja and Ferreira, 2008; Zhang, 2021). Thus, gaining a comprehensive understanding of teachers’ perspectives and standards becomes pivotal for promoting academic achievement. Within EFL contexts, a critical concern lies in the cultivation of resilience among teachers as they confront various challenges (Dewaele and Wu, 2021; Liu and Chu, 2022).

The notion of resilience which pertains to one’s capacity to rebound and move forward after facing challenges has been recognized as a significant factor in promoting teacher well-being and job satisfaction (Fried and Chapman, 2012). Put another way, it is the capacity to adjust to challenging conditions and boost one’s proficiency or skill when dealing with pressure and traumatic events (Bobek, 2002). It is a mental concept in education that has a significant impact on both instructors and students (Gu and Day, 2013). As noted by Hong (2012), one of the best methods to reduce the rate of EFL instructors’ quitting their jobs is to increase their resilience using the right techniques.

The second variable under investigation in this study is self-compassion. Self-compassion has been shown to be an important resource for coping with stress and adversity (Neff, 2003b) and has been related with reduced burnout and increased job satisfaction in instructors (Neff, 2011). Mindfulness and self-compassion are suggested to be effective strategies for reducing stress among instructors (Jennings et al., 2017). Self-compassion can help people to stay happily present even in painful situations, by keeping a healthy attitude towards personal flaws and avoiding dwelling on setbacks, rejection, or shame (Neff, 2003a; Allen and Leary, 2010). Individuals who practice self-compassion are less likely to feel threatened and more capable of handling difficult circumstances (Chishima et al., 2018; Tandler et al., 2019), which can improve their ability to cope with stress.

Teaching inevitably involves dealing with emotions. It is crucial for teachers to use successful coping mechanisms to regulate their feelings, given the undeniable importance of workplace emotions for teachers’ effectiveness (Deng et al., 2022). Teachers are able to assess and alter the duration and intensity of emotional events in the classroom using the concept of emotion regulation (Chang and Taxer, 2021). Koole (2009) defines emotion regulation (ER) as the range of techniques employed by individuals to manage their emotional states, including specific emotions, mood, affect, and stress. ER is crucial in L2 classes, in which educators must continuously regulate their feelings due to a variety of irritations and anxieties (Morris and King, 2018). It appears that L2 instructors cannot foster a positive learning environment in their classrooms unless they have control over their feelings, especially the negative ones (Namaziandost et al., 2022). This capacity to regulate emotions is a crucial socioemotional quality for EFL teachers, as it fosters resilience and adaptability in the face of challenging teaching circumstances (Wijaya, 2021). To put it another way, it is a technique that can enhance, sustain, and lessen the frequency, strength, and process of both positive and negative feelings (Koole, 2009).

Emotional labor, the last variable under research in this study, is defined by Hochschild (1983) as “the management of feeling to create a publicly observable facial and bodily display” (p. 7). It is a term frequently employed to explain how people alter their emotional expressions from their actual, felt feelings in order to communicate (Wang et al., 2019). Moreover, the endeavor, planning, and control required for teachers to exhibit organizationally wanted feelings during their interpersonal conversations with pupils as well as others in classroom and school settings is referred to as the emotional labor of teaching (Morris and Feldman, 1996; Yin et al., 2013). Despite the fact that instructors may show genuine emotion in front of the class, they frequently pretend to feel something they do not in order to help or hinder the growth of their students. This behavioral aspect of emotion regulation, known as emotional labor, represents the gap between felt and displayed emotions and may affect teachers’ psychological, behavioral, and physical adjustment (Taxer and Frenzel, 2015; Wang et al., 2019). Emotional labor strategies, which involve the effort required to regulate one’s emotions to meet job demands, have been recognized as a crucial source of stress and burnout in teachers (Wang et al., 2021), but their potential impact on teacher resilience has received less attention. Effective emotional labor strategies, including surface acting or deep acting, have been shown to reduce emotional exhaustion and promote job satisfaction in teachers (Burić and Frenzel, 2021).

Some studies (e.g., Yonezawa et al., 2011; Pena et al., 2012; Razmjoo and Ayoobiyan, 2019) on teacher resilience and its potential predictors have been performed in response to the importance and value of this factor in L2 education; however, it still needs more investigation to broaden the existing literature. In other words, there is still a lack of consensus on the most important predictors of resilience in teachers, particularly in the EFL context. In addition, existing studies have primarily focused on individual-level factors, such as personality traits or coping styles, and have paid less attention to job-related factors, such as emotional labor and regulation strategies, that may also impact teacher resilience (Ainsworth and Oldfield, 2019; Beltman, 2021). In this study, our aims were designed to explore the effects of teacher self-compassion, emotion regulation, and emotional labor strategies on teacher resilience within the EFL context. Having examined these variables and their relationships, we aim to contribute to the existing knowledge base on teacher resilience and provide valuable insights for educational practitioners specifically working in EFL settings. Understanding the predictors of teacher resilience in the EFL context is essential for the development of targeted interventions and support systems that can enhance teacher well-being and ultimately improve the quality of education in these unique language learning environments.

## Literature review

### Resilience

Gu and Day (2007) describe teacher resilience as the capacity of a teacher to recover and restore their capabilities or morale quickly and efficiently in challenging situations. Additionally, Mansfield et al. (2012) define this construct as a dynamic process where a teacher’s personal traits interact with contextual resources to shape their responses when dealing with adverse events. Although there is no consensus on the definition of teacher resilience, scholars generally agree that it involves a teacher’s ability to respond effectively to challenges (Clarà, 2017). Due to the importance and worth of teacher resilience in instruction, a number of researchers have examined the causes and effects of this construct (e.g., Mansfield and Beltman, 2019; Razmjoo and Ayoobiyan, 2019; Xie, 2021).

Regarding self-efficacy, teacher resilience was examined by Razmjoo and Ayoobiyan (2019). In doing so, two closed-ended surveys were distributed to 92 EFL instructors. The analysis of the participants’ answers revealed a strong and advantageous link between teachers’ self-efficacy and resilience. Likewise, Xie (2021) investigated the link between teachers’ emotional regulation and resilience. To do this, 314 Chinese teachers were given copies of two valid scales measuring the emotional control and resilience of instructors. The results of the correlational analysis showed a connection between teachers’ emotional regulation and resilience. Moreover, Neff (2003a) carried out a study and asserted that self-compassion was discovered to have a substantial positive correlation with life satisfaction and a substantial adverse relationship with anxiety and depression. This raises the possibility that practicing self-compassion is an adaptive process that improves psychological resiliency and wellbeing. Additionally, Lefebvre et al. (2020) indicated that developing a self-compassionate mindset is essential to fostering employee resilience at work, and that a variety of factors, including contemplative trainings, guidance and listening approaches, and individual factors, can enhance self-compassion in organizations. In another study by Bluth et al. (2018), results showed a strong favorable relationship between self-compassion and both resilience and curiosity. Adolescents who are more compassionate toward themselves are more resilient and recover more quickly from setbacks. Moreover, Hascher et al. (2021) carried out a study and reported a positive relationship between well-being and resilience.

The difficulties of establishing and sustaining teacher resilience, in addition to its indicators and effects, have also been investigated. For instance, Gu and Day (2013) investigated the issues affecting teacher resilience and discovered that the relational, personal, and organizational contexts in which they operate may have a negative impact on their resilience. They also found that maintaining and developing teacher resilience may be hampered by the socioeconomic environment of the workplace. In a similar vein, the challenges of fostering and sustaining resilience among instructors in Malaysian secondary institutions were examined by Razak (2013). To determine the difficulties and obstacles to their resilience, 46 Malaysian instructors were interviewed. The main challenges to maintaining teacher resilience were found to be a few contextual, personal, financial, and administrative problems.

Different traits that define resilience have been identified in the literature that is currently accessible. According to Tait (2008), resilient teachers frequently report feeling highly satisfied with their work, react favorably to stressful situations, demonstrate useful coping mechanisms, and are extremely effective and emotionally intelligent educators. Additionally, Howard and Johnson (2004) came to the conclusion that resilient instructors show a sense of autonomy, possess behavioral management competencies, can minimize unpleasant emotions, sympathize with their students, have moral goals, and are skilled and encouraging. Besides, Taylor (2013) suggested that instructors with great resiliency have a malleable control system, liberty, positivity, dedication, a positive rapport, and an appreciation of pedagogical changes. Khanshan and Yousefi (2020) asserted the same thing, arguing that resilient instructors are self-assured, optimistic, able to form strong bonds with others, inspired, proficient, and sensitive to crucial events. As stated by Day and Gu (2013), teachers who are resilient have long-lasting efficacy and dedication.

### Self-compassion

The concept of self-compassion, introduced by Neff (2003b), draws on Buddhist ideas and serves as an alternative to self-esteem. Compassion involves acknowledging and being responsive to others’ suffering without avoiding or disconnecting from it. In contrast to the Western view that compassion is primarily directed towards others, Eastern philosophies like Buddhism do not differentiate between self and others. Self-compassion entails being open to one’s own pain, showing kindness and understanding to oneself instead of self-criticism, recognizing that one’s experiences are part of the human experience, and holding painful thoughts and emotions in balanced awareness. To provide a comprehensive definition of self-compassion, Neff (2003b) identifies three key components: self-kindness, common humanity, and mindfulness.

According to research by Neff et al. (2007), self-reported measures of happiness, positivity, positive affect, intelligence, personal initiative, interest, and inquiry, life satisfaction, extroversion, and conscientiousness all showed a significant positive relationship with self-compassion. According to some researchers, compassion is a type of emotion that is different from other feelings that are comparable to it, such as love, empathy, sorrow, or grief (Goetz et al., 2010). Others see this notion as a mindset, suggesting that people deliberately choose to think compassionately (Sprecher and Fehr, 2005). There are numerous aspects of compassion, including cognitive, emotional, purposeful, and motivational ones (Jazaieri et al., 2013). Despite the disagreements over what exactly constitutes compassion, most people concur that it entails a general awareness of another person’s suffering combined with a desire to provide assistance in some way (Sprecher and Fehr, 2005; Jazaieri et al., 2013).

Studies have demonstrated that self-compassion has a positive impact on well-being and satisfaction with life (Raes, 2010; Baker and McNulty, 2011). By reducing feelings of threat and enhancing control over stressful situations (Chishima et al., 2018), self-compassion enables the use of effective stress-coping techniques (Tandler et al., 2019). Moreover, self-compassion safeguards the ego against self-criticism, promotes motivation for self-improvement and performance enhancement, and encourages making amends when necessary (Breines and Chen, 2012). It appears that greater acceptance of one’s imperfections increases the likelihood of taking corrective action. Instructors who are self-compassionate are more helpful, happier, and experience greater professional achievement (Moè and Katz, 2020). In fact, research suggests that self-compassion may be linked to effective and adaptive emotion regulation (Diedrich et al., 2014).

Self-compassion differs from self-esteem. Having self-esteem requires assessing one’s worth (Neff, 2011). Analysis of the study on self-esteem reveals a decrease in its appeal, which may be caused by its link to exaggerated self-perceptions or a general obsession with the self (Neff, 2011). When practicing self-compassion, the emphasis is on providing helpful responses to hardship or making links to the experiences of others rather than on evaluating oneself or comparing oneself to others (Neff, 2003b). In this regard, Leary et al. (2007) discovered that self-compassionate individuals did not replay embarrassing or humiliating situations in their thoughts when they were faced with failure, rejection, or humiliation. Instead, they analyzed their flaws and acknowledged the role they played in how things turned out.

### Emotion regulation

The physiological, behavioral, and cognitive processes that people use to efficiently control and react to emotional experiences are referred to as emotion regulation (Gross and Thompson, 2007). That is, people’s ability to regulate their emotions affects the types of emotions they encounter, when they feel them, as well as how they convey them. Experienced emotions are controlled to achieve educational goals, like other kinds of self-regulation and self-management skills. To do this, a variety of strategies—referred to as emotion regulation strategies—may be used (Taylor et al., 2020; Li and Liu, 2021). Both instructors and students encounter a range of emotional situations in the classroom. As the focal point of the classroom, instructors are expected to create an ideal emotional environment in which they should control both their own and their students’ feelings (Taxer and Gross, 2018; Deng et al., 2022). In other words, emotion regulation provides both instructors and pupils with the ability to strengthen positive emotions while reducing negative ones (Fried, 2011).

Emotion regulation is regarded as an interpersonal endeavor that is linked to a person’s ability in controlling how and when they should perceive and express feelings (Gross, 1998). Emotion regulation techniques are frequently used by effective pupils and instructors in the instructional realm (Shafiee Rad and Jafarpour, 2022). Up-regulating positive feelings is a technique used in L2 emotion regulation to make learning more pleasant, handle academic duties effectively, and increase their effectiveness (Zhang et al., 2020). Through efficient interpersonal relations, effective teaching, and pupils’ accomplishments in the learning process, pupils and instructors in L2 education can skillfully control their negative and positive feelings (Teng and Zhang, 2016; Bing et al., 2022; Shafiee Rad and Jafarpour, 2022). Instructors are better able to handle difficulties that arise during the teaching/learning process and propose remedies by regulating their emotions (Greenier et al., 2021; Thoma, 2021; Zhao, 2021).

Earlier studies in the domain of emotion regulation have demonstrated the prevalence and major contributions of teacher emotion regulation to successful instruction. For example, Shafiee Rad and Jafarpour (2022) carried out a study and concluded that well-being, grit, emotion regulation, and resilience can greatly enhance L2 learners’ writing abilities. In conclusion, they asserted that using positive emotion interventions can enhance people’s learning abilities as well as their feelings. Besides, Morris and King (2018) examined how effectively emotion regulation techniques helped university language instructors deal with their frustration during class. They discovered that instructors at language universities used context-dependent emotion regulation techniques that helped them feel more in control and confident in the face of stress. Similarly, the purpose and effectiveness of emotion regulation in classrooms were examined by Taxer and Gross (2018). They came to the conclusion that educators with hedonic and instrumental emotion regulation objectives attempted to control their own emotions as well as those of their students. Besides, the use of teacher emotion regulation techniques in reaction to student misbehavior was examined in recent research by Chang and Taxer (2021). They came to the conclusion that instructors who regularly reappraise are less likely to feel unpleasant emotions in the presence of pupils’ misbehavior and show less repression when unpleasant emotions are felt. The methods applied to regulate emotions were categorized as reappraisal or suppression.

### Emotional labor

Teachers are required to regulate their emotions by suppressing negative ones and amplifying positive ones, while also avoiding displays of excessively strong or weak emotions, regardless of their valence (Burić and Frenzel, 2021). Additionally, they are expected to display enthusiasm and passion, and use emotional displays to enhance their teaching effectiveness, maintain professionalism, and manage student misbehavior (Winograd, 2003; Taxer and Gross, 2018). To meet these expectations, teachers often engage in emotional labor, which involves regulating their internal and external emotional experiences in accordance with the emotional display norms of their professional roles (Yin and Lee, 2012; Taxer and Frenzel, 2015; Burić and Frenzel, 2021). Hochschild (1983) defines emotional labor as an individual’s effort to regulate the components of their emotions in accordance with the emotional display standards of their professional roles. Wang et al. (2021) asserted that emotional labor is positively connected to well-being.

Moreover, emotional labor (EL), as described by Arlie Hochschild (1983), is “the management of feeling to create a publicly observable facial and bodily display” (p. 7). In the teaching context, emotional labor is mainly understood as the procedure by which instructors attempt to suppress, produce, and control their feelings and emotional expression in accordance with the moral beliefs and standards held about the teaching profession (Yin and Lee, 2012). Teachers must use specific techniques to regulate their emotions and feelings while working in order to teach EL successfully. Surface acting and deep acting are the two most commonly mentioned classical tactics in EL studies (Hochschild, 1983; Wharton, 2009).

Prior studies have suggested two primary emotional labor strategies: deep acting and surface acting. Deep acting is the deliberate management of interior emotions through the intentional engagement in ideas and actions that promote the experience and manifestation of the necessary emotion. Deep acting results in the experience and sincere display of acceptable feelings as specified by emotional rules (Burić and Frenzel, 2021). Surface acting, on the other hand, is concerned with the direct alteration of one’s apparent expression to match with the needed mood (Brotheridge and Grandey, 2002). As a result, there is a mismatch between the perceived emotion and the emotion communicated (Grandey, 2015). Surface acting has been defined as hiding or repressing one’s true emotions, as well as simulating necessary emotional responses.

When it comes to instructors’ emotional regulation, some circumstances may necessitate hiding specific emotions they consider insufficient. Instructors, for example, may wish to hide excessive concern for a student in order to avoid being biased or laughter at a poor student joke in order to keep their control (Taxer and Frenzel, 2015). In turn, other circumstances necessitate instructors acting out feelings such as anger in order to maintain classroom management or excitement to increase student engagement (Sutton et al., 2009).

### The hypotheses of the study

*H1:* Teacher self-compassion directly predicts teacher resilience.

Some studies have documented a positive relationship between teacher self-compassion and resilience (Nery-Hurwit et al., 2018; Chen, 2022). Self-compassion is concerned with treating oneself kindly, recognizing one’s shared humanity with others, and being mindful of one’s experiences. These qualities can help instructors cope with the many stressors and challenges they face in their work, and may promote greater resilience in the face of adversity. For example, Sauve (2017) reported that teacher self-compassion was associated with resilience, teacher efficacy, and burnout. Theoretical background supports this hypothesis, as self-compassion has been associated with further resilience, mindfulness, well-being, and classroom quality (e.g., Jennings, 2015; Kotera et al., 2021; Demetriou et al., 2023).

*H2:* Teacher self-compassion directly affects teacher emotional labor strategies.

From a theoretical viewpoint, self-compassion may help instructors manage the emotional demands of their job, potentially reducing the emotional burden of teaching (Newcomb, 2021). Self-compassion may also promote greater well-being and job satisfaction, which could mitigate the negative effects of emotional labor (Hwang et al., 2019; Moè & Katz, 2020) or help them to use adaptive emotional labor strategies.

*H3:* Teacher emotion regulation directly affects teacher emotional labor strategies.

Effective emotion regulation can help teachers manage the emotional demands of their work, using their emotional labor strategies more effectively (Ye and Chen, 2015; Burić, 2019; Xie et al., 2022). For example, teachers who use adaptive emotion regulation strategies (e.g., reappraisal) are better able to manage emotional labor than those who use maladaptive strategies (e.g., suppression). This hypothesis is supported by Ghanizadeh and Royaei (2015) who found that emotion regulation was significantly associated with emotional labor strategies.

*H4:* Teacher emotion regulation directly affected teacher resilience.

In light of Theoretical and empirical literature, it can be hypothesized that emotion regulation can directly affect resilience. Effective emotion regulation can help individuals maintain a positive outlook, manage stress, and bounce back from adversity (Beltman et al., 2011; Beltman, 2021). Teachers who are better able to regulate their emotions may be better equipped to handle the many stressors and challenges they face in their work, and may be more resilient as a result (Gu and Day, 2007, 2013; Li and Lv, 2022).

*H5:* Teacher emotional labor strategies directly affect teacher resilience.

The relationship between emotional labor and resilience is also supported in the literature (Yin et al., 2013; Hyun-Ji and Hyunkyung, 2017; Wang et al., 2019; Yang Y. et al., 2022). Using effective emotional labor strategies can be a significant source of managing stress and burnout for teachers, helping teachers cope with stressors and maintain a positive outlook. Emotional labor may also contribute to other negative outcomes, such as teacher burnout and turnover intention (Cheung et al., 2011; Yin et al., 2019). As such, reducing emotional labor may be an important strategy for promoting teacher well-being and resilience (McKay and Barton, 2018).

### The present study

In this research, our primary objective is to investigate the influence of teacher self-compassion, emotion regulation, and emotional labor strategies as predictors of teacher resilience in the unique context of EFL instruction. EFL teaching poses distinct challenges for educators as they navigate linguistic barriers, cultural diversity, and varied learning needs among their students (Johnston, 1997; Lee, 2010). Beyond simply teaching English, EFL teachers are responsible for creating a supportive and engaging environment that nurtures language acquisition and cultural understanding (Murphy, 1988; Chen and Goh, 2011; Fathi et al., 2023). Managing emotions effectively, cultivating resilience, and handling the emotional demands inherent in this context are crucial for EFL instructors to deliver quality education and address the diverse needs of their students (Yang S. et al., 2022). Moreover, EFL teachers frequently encounter situations where emotional regulation and the use of emotional labor strategies become necessary (Aragão, 2011; Greenier et al., 2021). They may need to conceal their genuine emotions to maintain a positive classroom atmosphere, motivate students, and foster inclusivity (Dewaele, 2015). Understanding how self-compassion, emotion regulation, and emotional labor strategies function within the specific context of EFL teaching is pivotal in developing targeted interventions and support systems that enhance teacher resilience and overall well-being. By examining these factors in the EFL context, our study contributes valuable insights into the dynamics and predictors of teacher resilience within this distinct educational setting. Furthermore, the findings from this research will inform the development of contextually relevant training programs and interventions that specifically address the challenges faced by EFL teachers, ultimately elevating their professional practice and well-being. Through a comprehensive exploration of the objectives and significance of the EFL context within our study, we aim to provide a clearer understanding of its contextual importance and contribute to the existing body of literature on teacher resilience in EFL instruction.

## Method

### Participants

The study recruited a sample of 711 Chinese EFL teachers including 398 female (56%) and 3,131 male (44%) English teachers. The average age of the participants was 38 years, with a range from 23 to 65 years. Concerning teaching experience, about 30% of the participants had less than 5 years of experience, 44% had 5–15 years of experience, and 25% had more than 15 years of experience. As far as educational background was concerned, 41% of the participants had a bachelor’s degree in mostly in English majors, 35% have a master’s degree, and about 23% have a doctoral degree in an English related field. Regarding regional distribution of the sample., about 20% of the participants are from the Eastern region of China, 24% are from the Central region, 30% are from the Western region, and 24% are from the Northern region. Table 1 illustrates the demographic information of the participants.

**Table 1:** Demographics of participants (N = 711).

Demographic variable	Frequency	Percentage
Gender		
Female	398	56
Male	313	44
Age		
Mean	38	
Range	23–65	
Teaching experience		
< 5 years	219	30.80
5–15 years	314	44.16
> 15 years	178	25
Educational background		
Bachelor’s degree	293	41.20
Master’s degree	249	35
Doctoral degree	169	23.76
Region		
Eastern	148	20.8
Central	175	24.61
Western	216	30.37
Northern	172	24.1

### Instruments

The Teacher Resilience Scale was utilized in this study to measure the resilience of teachers. The Resilience Scale, which consists of 14 items, was originally developed by Wagnild and Young (1993) and has demonstrated high reliability and validity. In this study, the English version of the scale was used, which has five subfactors: meaning and purposeful life, perseverance, equanimity, self-reliance, and existential aloneness. Participants rated each item on a 7-point Likert scale, ranging from 1 (strongly disagree) to 7 (strongly agree). In this study, the reliability of the Resilience Scale was assessed using Cronbach’s alpha, which was found to be 0.83.

The Self-Compassion Scale (SCS; Neff, 2003a) evaluates individuals’ compassionate attitudes towards themselves when facing challenges or hardships. The scale assesses the extent to which individuals can offer themselves care and understanding when acknowledging their flaws, instead of criticizing or neglecting themselves. It also measures individuals’ ability to recognize that suffering and failure are common to human experience, and to manage negative emotions in a balanced way. Respondents rate 26 items on a five-point Likert scale, ranging from 1 (almost never) to 5 (almost always). The SCS comprises six subscales: self-kindness, self-judgment, common humanity, isolation, mindfulness, and over-identification. For this study, a composite score of self-compassion was computed by averaging the scores of the self-judgment, isolation, and over-identification subscales (reverse scored). The reliability coefficient of the scale was evaluated using Cronbach’s alpha, which was reported to be 0.79 in this research.

The study assessed emotional labor strategies of teachers using the Teacher Emotional Labor Strategy Scale (TELSS) developed by Yin (2012). The TELSS has three subscales: surface acting (five items), deep acting (three items), and genuine expression (three items). Respondents rated their level of agreement on a seven-point Likert scale, ranging from one (strongly disagree) to seven (strongly agree). The internal consistencies of the scale were found to be acceptable in a previous study with Chinese teachers (Yin, 2012), with coefficients of 0.84 for surface acting, 0.70 for deep acting, and 0.67 for genuine expression. In this study, only the items from the deep acting and genuine expression subscales were employed. In this study, the reliability coefficients of these two sub-scales were reported to be 0.82 and 0.85, respectively.

To evaluate the emotional regulation of EFL teachers, the researchers utilized the emotion regulation scale created by Gross and John (2003). The scale consists of 10 items and measures the respondents’ inclination and methods for regulating their emotions in two areas: (1) Cognitive Reappraisal and (2) Expressive Suppression. Participants were asked to use a 7-point Likert-type scale varying from 1 (strongly disagree) to 7 (strongly agree) to answer each item.

### Procedure

The study used an online survey to collect data from the participants. They were invited to complete the survey voluntarily, and informed consent was obtained before the survey. The survey consisted of two sections: demographic information and the four self-report measures. The demographic information included gender, age, educational background, teaching experience, and region of teaching. The participants accessed the survey using their personal computers or smartphones through online platforms and professional networks. The study assured the participants that their participation was confidential, and they could give up from the study at any time. The data collection process lasted for 1 month.

### Data analysis

Firstly, the data was examined for adherence to fundamental assumptions, such as case-to-variable ratios, normality, linearity, missing data, and outliers (Tabachnick et al., 2013; Kline, 2016). Descriptive statistics and variable reliability were then calculated using SPSS 23.0. The instruments’ psychometric properties were assessed by conducting confirmatory factor analysis (CFA), refining the model by evaluating the overall fit of the measurement model and the ability of individual items to define their assigned latent factors. Structural equation modeling (SEM) was subsequently employed to test the study’s hypotheses by analyzing the relationships’ structural coefficients. The model fit was evaluated using the maximum likelihood estimation method, with values less than three indicating an acceptable data-model fit for relative chi-square (Tabachnick et al., 2013). For comparative fit indices (CFI) and Tucker-Lewis index (TLI), values greater than 0.90 were deemed acceptable, while root mean square error of approximation (RMSEA) and standardized root mean square residual (SRMR) values less than 0.06 were considered a close fit (Hu and Bentler, 1999).

### Results

Before proceeding with the primary analyses, initial analyses were performed to verify that the data satisfied key assumptions. Missing data, univariate and multivariate outliers, and non-normality were dealt with. SPSS 23.0 was used to determine the data’s distribution and identify any potential outliers through descriptive statistics and reliability analysis. To identify univariate outliers, the *z*-scores of each variable were examined, while Mahalanobis distance values were used to detect multivariate outliers (Kline, 2016). The expectation–maximization algorithm was used to manage missing data. Skewness and kurtosis values were used to assess normality, while scatter plots of the independent and dependent variables were used to examine linearity. Additionally, Mardia’s value, a measure of multivariate skewness and kurtosis, was calculated to further assess the multivariate normality of the data (Mardia, 1970).

Table 2 presents the means, standard deviations, and correlations among the study variables. The results showed that all the correlations among the variables were significant at the *p* < 0.01 level, indicating that there were significant relationships among the study variables. The mean scores for teacher self-compassion, emotion regulation, emotional labor, and teacher resilience were 3.37 (SD = 0.63), 4.07 (SD = 0.80), 3.46 (SD = 0.56), and 3.73 (SD = 0.60), respectively.

**Table 2:** Descriptive statistics and correlations.

	*M*	*SD*	1	2	3	4
1. Teacher self-compassion	3.37	0.63	1			
2. Emotion regulation	4.07	0.80	0.44**	1		
3. Emotional labor	3.46	0.56	0.37**	0.42**	1	
4. Teacher resilience	3.73	0.60	0.46**	0.54**	0.45**	1

*M*, mean; SD, standard deviation; **p < 0.01.

Confirmatory factor analysis was conducted to assess the validity of the scales used in the study. The results showed that the four-factor model had a good fit to the data, as indicated by the following fit indices: χ^2^ (478) = 1069.42, *p* < 0.001; RMSEA = 0.05; CFI = 0.96; TLI = 0.95. The factor loadings for all the items were significant at the *p* < 0.001 level, ranging from 0.49 to 0.90, indicating good convergent validity.

Once the measurement model was confirmed, various structural models were assessed to verify the hypotheses. The partial mediation model (Model C) was compared with the full mediation model (Model B) and the direct model (Model A). Table 3 presents the fit statistics for all three models. The results revealed that the hypothesized model (Model C) had a significantly better fit compared to the other models. Therefore, Model C was deemed the most parsimonious fit for the data.

**Table 3:** Results of fit indices of structural models.

Model	χ^2^	df	GFI	CFI	RMSEA	TLI	SRMR
Direct effect model (A)	1174.420**	625	0.82	0.90	0.06	0.89	0.18
Full mediation model (B)	949.185**	621	0.87	0.96	0.04	0.94	0.07
Partial mediation model (C)	867.246**	616	0.90	0.97	0.03	0.96	0.06

Δχ^2^ shows differences between model and the subsequent model; **p-value < 0.001.

**Figure 1:**
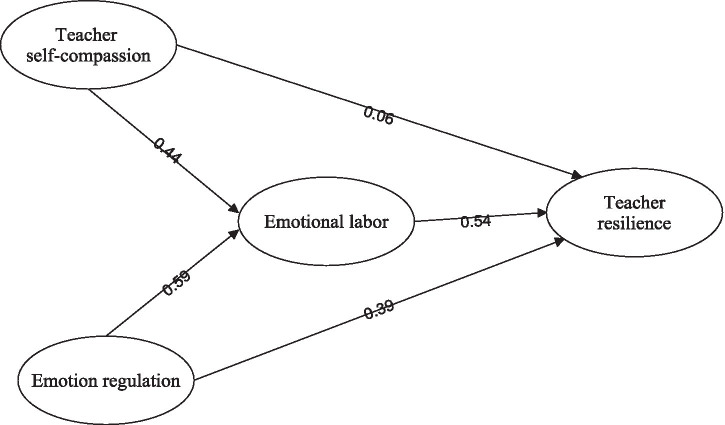
The final fit model.

The final fitted model’s path and parameter estimates are presented in Figure 1. As illustrated in Figure 1, all path coefficients were statistically significant, except for the relationship between teacher self-compassion and resilience. The structural model revealed that teacher self-compassion significantly affected teacher emotional labor (*β* = 0.44, *p* < 0.01), and teacher emotion regulation had a significant positive influence on teacher emotional labor (*β* = 0.59, *p* < 0.01). Moreover, teacher emotional labor was positively associated with resilience (*β* = 0.54, *p* < 0.01).

Next, the study used Baron and Kenny’s (1986) method to examine whether teacher emotional labor acted as a mediator between the variables. The direct model (displayed in Table 4) indicated significant path coefficients between teacher self-compassion, teacher emotion regulation, and resilience (self-compassion → resilience: 0.17, *p* < 0.05; emotion regulation → resilience: 0.42, *p* < 0.001), confirming the first step of Baron and Kenny’s method. The full mediation model showed that self-compassion and emotion regulation significantly influenced emotional labor (self-compassion → emotional labor: 0.37, *p* < 0.001; emotion regulation → emotional labor: 0.62, *p* < 0.001), thus confirming the second step of the method. The partial mediation model indicated that teacher emotional labor partially mediated the relationship between teacher self-compassion and teacher resilience. Furthermore, teacher self-compassion had an insignificant path coefficient on resilience, while teacher emotional labor acted as a complete mediator between teacher emotion regulation and resilience. Hence, the impact of self-compassion on teacher emotional labor impacted resilience.

**Table 4:** Path estimates of structural model.

Standardized path coefficients (*t*-value)			
	Direct effects model	Full mediation model	Partial mediation model
SC → resilience	0.17 (2.79*)		0.06 (0.47)
ER → resilience	0.42 (4.57***)		0.39 (3.98**)
SC → emotional labor		0.37 (4.54**)	0.44 (5.37***)
ER → emotional labor		0.62 (8.34***)	0.59 (7.88***)
Emotional labor → resilience		0.59 (7.27***)	0.54 (6.08***)

SC, self-compassion; ER, emotion regulation; *p-value < 0.05; **p-value < 0.01; ***p-value < 0.001.

## Discussion

The current study sought to explore the relationship between teacher self-compassion and emotion regulation as predictors of teacher resilience in EFL context via the mediating role of emotional labor. First of all, the results of this study indicated that teacher self-compassion directly predicted teacher resilience. This finding is in accordance with previous research that has emphasized the importance of positive relationships between individuals’ self-compassion and their resilience (e.g., Neff, 2003a,b; Bluth et al., 2018; Nery-Hurwit et al., 2018; Lefebvre et al., 2020; Kotera et al., 2021; Chen, 2022). It can be argued that teacher resilience at work requires a self-compassionate attitude, and higher self-compassion is linked to greater resilience among teachers. From this perspective, instructors who were more self-compassionate were much more resilient; they recovered more quickly from setbacks; they were more tolerant and understanding and faced less anxiety when they failed to fulfill their moral preferences. One potential cause for the association between self-compassion and resilience is that the mindfulness component of self-compassion helps people stay focused on difficult situations, allowing them to respond constructively instead of worrying or responding irrationally (Bluth et al., 2018). Previous studies have shown that self-compassion is associated with higher levels of resilience in various populations, including healthcare workers (Benzo et al., 2017), college students (Smeets et al., 2014), and adults with chronic pain (Edwards et al., 2019). In the context of teaching, self-compassion has been found to be positively associated with emotional stability and job satisfaction (Jennings, 2015), and negatively associated with stress (Hwang et al., 2019). Theoretically, self-compassion is regarded as an adaptive response to the emotional and physical demands of instruction. By treating oneself with kindness and understanding, teachers may be better able to cope with the stress and emotional demands of their work. Self-compassion may also promote a sense of self-efficacy and control (Liao et al., 2021), which can contribute to greater resilience in the face of adversity.

The second finding of this study was that teacher emotional labor strategies directly predicted teacher resilience. This finding is partially in line with studies by Wang et al. (2021) who reported a positive relationship between emotional labor and well-being. It was found that teachers with higher emotional labor competence, had higher levels of resilience while facing various challenges, resulting in a more positive teaching environment. One justification for this finding can be due to the fact that teachers have a crucial role in setting up a positive learning environment; therefore, by using various strategies of emotional labor, their resiliency will increase, and as a result, they will be better able to cope with the difficulties of the teaching profession and classrooms, boost learners’ achievements, and enhance the learning environment. Emotional labor is concerned with the effort that individuals expend to manage their emotions during interactions with others (Hochschild, 1983). In the educational contexts, emotional labor involves managing one’s emotions in response to students, colleagues, and parents, and includes strategies such as surface acting (i.e., faking emotions) and deep acting (i.e., modifying one’s emotions to align with one’s true feelings) (Burić and Frenzel, 2021). Previous research has indicated that emotional labor strategies can have effects on teacher well-being and job satisfaction (Cheung et al., 2011; Kinman et al., 2011). Also, the present finding suggests that emotional labor strategies may also be a positive resource for promoting teacher resilience. This finding is consistent with previous research that has highlighted the adaptive nature of emotional labor strategies (Diefendorff et al., 2005; Yin et al., 2013). Emotional labor strategies may be seen as a form of emotion regulation, which is associated with resilience (Fried and Chapman, 2012). By managing their emotions in response to the demands of their work, teachers may be better able to maintain a sense of control and self-efficacy, which can contribute to greater resilience in the face of adversity (Liu and Chu, 2022; López-Angulo et al., 2022).

The last finding of this study was that teacher emotion regulation indirectly predicted teacher resilience via the mediation of emotion labor strategies. This finding is partially in line with previous research (e.g., Xie, 2021; Li and Lv, 2022; Shafiee Rad and Jafarpour, 2022) that highlight the significant relationship between these two constructs in particular domains and contexts. It was revealed that when instructors are able to regulate their emotions, they are more likely to have control over their feelings as a result of their emotional labor strategies, which in turn enhances their resilience. One likely justification for this finding can be the fact that when instructors are able to correctly hide their feelings while facing difficulties or intensify their emotions while trying to boost learners’ engagement and achievement, this will result in higher teacher resilience, which in turn provides instructors with higher levels of control over both their feelings and classroom management.

The present study suggests that emotion regulation may promote resilience by facilitating the development of effective emotional labor strategies. This finding is consistent with previous research that has highlighted the role of emotion regulation in promoting adaptive coping strategies (Aldao et al., 2010; Bonanno and Burton, 2013). This finding implies that emotion regulation might play a critical role in promoting resilience among teachers by facilitating the development of effective emotional labor strategies. By regulating their emotions in reaction to different demands and stressors in their work, educators may be better able to develop and employ adaptive emotional labor strategies, which in turn may enhance their resilience. Moreover, this finding is also consistent with the theoretical perspective of the transactional model of stress and coping (Lazarus and Folkman, 1984), which emphasizes the importance of the interaction between environmental demands and an individual’s coping resources. According to this perspective, individuals with effective coping resources, such as emotion regulation skills, are better able to cope with stressful situations and maintain resilience (Lazarus and Folkman, 1987).

The findings of this study offer valuable insights into the EFL instructional context and its implications for teacher resilience. The initial finding emphasizes the positive association between teacher self-compassion and resilience, aligning with previous research that underscores the significance of self-compassion in fostering resilience. Given the language barriers and diverse student needs in the EFL context (Lee, 2010; Joe and Lee, 2013), self-compassion plays a pivotal role in managing the emotional demands of teaching. By practicing self-kindness and understanding, teachers can effectively cope with stress, overcome setbacks, and maintain a positive outlook. The second finding highlights the direct link between emotional labor strategies and teacher resilience. In EFL instruction, emotional labor is essential for cultivating a positive learning environment and effectively navigating interactions with students, colleagues, and parents (Aragão, 2011; Dewaele and Wu, 2021). Skillful utilization of emotional labor strategies enhances teacher resilience by enabling them to address challenges and foster a supportive teaching environment. The third finding reveals that teacher emotion regulation indirectly predicts resilience through the mediating effect of emotional labor strategies. By regulating their emotions, EFL teachers gain control over their feelings and can employ effective emotional labor strategies, thereby contributing to higher levels of resilience. Overall, these findings not only underscore the contextual relevance of EFL instruction but also deepen our understanding of the dynamics of teacher resilience within this unique educational setting.

## Conclusion

This investigation was carried out to probe into the impact of teacher self-compassion and emotional regulation as predictors of teacher resilience in the Chinese EFL context via the mediating role of emotional labor. It was revealed that higher levels of self-compassion, emotional regulation, and emotional labor can result in higher teacher resilience, which is a crucial factor for instructors in order to maintain their control over the classroom and learners. Teachers sometimes need to hide their true feelings in order to keep the classroom atmosphere positive. On the other hand, they might need to intensify their emotions in order to encourage learners and motivate them to better engage in classroom activities. These findings highlight the importance of individual and job-related factors in promoting teacher resilience and well-being.

Concerning the theoretical implications, the present findings verify the growing literature on the importance of positive psychological resources, such as self-compassion, in promoting resilience and well-being. Also, they highlight the adaptive nature of emotional labor strategies and the potential for these strategies to contribute to resilience in the face of job-related stressors. In addition, the critical role of emotion regulation in promoting effective coping strategies and resilience is emphasized. The findings of this investigation have significant practical implications for teacher training programs and school-based interventions, aiming to enhance teacher resilience and mitigate burnout in the Chinese EFL context. Firstly, these programs can integrate strategies that foster self-compassion and improve emotion regulation skills among teachers. By nurturing self-compassion, instructors can cultivate a compassionate attitude towards themselves, facilitating better self-care and emotional well-being. Additionally, promoting effective emotion regulation strategies equips teachers with the necessary tools to navigate challenging situations and manage their emotions more effectively. Secondly, providing opportunities for teachers to develop and employ adaptive emotional labor strategies in response to job-related stressors can greatly contribute to their resilience. Recognizing the dual nature of emotional labor, EFL teachers can learn to strike a balance between concealing their true feelings to maintain a positive classroom atmosphere and intensifying their emotions to motivate and engage learners. Equipping EFL teachers with adaptive emotional labor strategies can enhance their resilience by effectively managing the emotional demands of their profession.

Moreover, fostering positive emotions and minimizing negative emotions within the classroom setting can have a profound impact on motivation, effort, and achievement in foreign language learning. By incorporating strategies that enhance positive emotions and reduce negative ones, teachers can create an optimal learning environment for their students, resulting in improved learning outcomes. Furthermore, these findings support and contribute to existing literature that emphasizes the significance of positive psychological resources, such as self-compassion, in promoting resilience and well-being. They underscore the adaptable nature of emotional labor strategies and their potential to bolster resilience when faced with job-related stressors. Additionally, the critical role of emotion regulation in fostering effective coping strategies and resilience is highlighted. Implementing the practical implications derived from this research can effectively support EFL instructors in their professional development as knowledgeable, compassionate, and resilient educators. Developing resilience not only enhances their commitment and inspiration but also equips them to effectively support students’ educational growth (Gu et al., 2015). Embracing the concept of resilience has the potential to transform EFL instructors into highly skilled professionals who are well-prepared to navigate diverse challenges and make positive contributions to teaching and learning initiatives.

Taken together, in EFL settings, where language barriers and diverse student needs are prevalent (Kalaja and Ferreira, 2008), cultivating self-compassion becomes essential for managing the emotional demands of teaching. By showing themselves kindness and understanding, teachers can effectively handle stress, bounce back from setbacks, and maintain a positive outlook. Additionally, the study underscores the importance of employing effective emotional labor strategies to create a supportive and inclusive learning environment while effectively navigating interactions with students, colleagues, and parents. EFL teachers who possess adaptive emotional labor strategies are better equipped to overcome challenges and cultivate a positive teaching atmosphere. These findings provide valuable insights for the development of teacher training programs and interventions tailored to EFL contexts, aiming to enhance teacher resilience and alleviate burnout.

The present study, like other research studies, has limitations. First, the study used only self-report quantitative measures, which may be subject to response biases. Second, the study was cross-sectional in nature, which limits the ability to draw causal conclusions about the relationships among the variables. Third, the study was conducted in a specific cultural and educational context of China, which may affect the generalizability of the results to other EFL settings.

## Data availability statement

The raw data will be accessed without any reservation upon request. Requests to access these datasets should be directed to YH, yajuzibisha317@163.com.

## Ethics statement

The studies involving human participants were reviewed and approved by School of Maxism, Sichuan International Studies University, Chongqing, 40031 China. The patients/participants provided their written informed consent to participate in this study.

## Author contributions

The author confirms being the sole contributor of this work and has approved it for publication.

## Funding

This work was supported by analysis of the impact of the ideology in colleges and universities (Item Number: sisu2019060).

## Conflict of interest

The author declares that the research was conducted in the absence of any commercial or financial relationships that could be construed as a potential conflict of interest.

## Publisher’s note

All claims expressed in this article are solely those of the authors and do not necessarily represent those of their affiliated organizations, or those of the publisher, the editors and the reviewers. Any product that may be evaluated in this article, or claim that may be made by its manufacturer, is not guaranteed or endorsed by the publisher.
